# (*E*,*E*,*E*,*E*)-2,3,5,6-Tetra­kis{2-[4-(dimethyl­amino)­phen­yl]ethen­yl}pyrazine

**DOI:** 10.1107/S1600536811019714

**Published:** 2011-05-28

**Authors:** Volker Schmitt, Dieter Schollmeyer, Heiner Detert

**Affiliations:** a University Mainz, Duesbergweg 10-14, 55099 Mainz, Germany

## Abstract

In the title compound, C_44_H_48_N_6_, the essentially planar mol­ecule [maximum deviation from the mean plane of the π system of 0.271 (3) Å] is located on a crystallographic centre of inversion. The almost planar (angle sums at N atoms = 357.6 and 357.1°) dimethyl­amino groups and short C—N bonds of the aniline groups [both 1.379 (4) Å] indicate strong electronic coupling between these groups and the central pyrazine ring.

## Related literature

For similar tetra­styryl­pyrazines prepared by acid- or base-catalysed condensations, see: Takahashi & Satake (1952 ▶). For photochemical properties of tetra­styryl­pyrazines, see: Collette & Harper (2003 ▶); Rumi *et al.* (2008 ▶) For star-shaped chromo­phores, see: Detert *et al.* (2010 ▶); Detert & Sugiono (2005 ▶); Strehmel *et al.* (2003 ▶); Nemkovich *et al.* (2010 ▶). For a two-dimensional homologue of the linear distyryl­pyrazine, see: Fischer *et al.* (2011 ▶). For cruciforms with a central benzene ring and phenyl­ene­vinyl­ene arms, see: Niazimbetova *et al.* (2002 ▶), Hauck *et al.* (2007 ▶), Zucchero *et al.* (2010 ▶). For probes for thrombine detection, see: Yan *et al.* (2011 ▶).
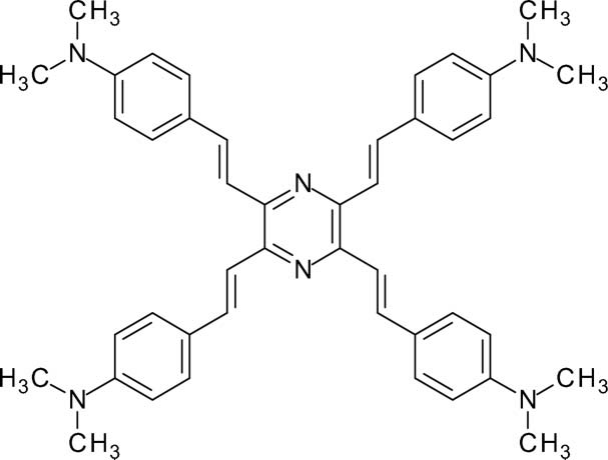


## Experimental

### 

#### Crystal data

C_44_H_48_N_6_
*M* *_r_* = 660.88
Monoclinic, 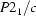

*a* = 10.763 (2) Å
*b* = 16.6751 (19) Å
*c* = 10.755 (3) Å
β = 101.109 (9)°
*V* = 1894.1 (7) Å^3^
*Z* = 2
Cu *K*α radiation
μ = 0.53 mm^−1^
*T* = 193 K
0.30 × 0.20 × 0.10 mm

#### Data collection

Enraf–Nonius CAD-4 diffractometer
3778 measured reflections
3584 independent reflections
2384 reflections with *I* > 2σ(*I*)
*R*_int_ = 0.073
3 standard reflections every 60 min  intensity decay: 2%

#### Refinement

*R*[*F* ^2^ > 2σ(*F* ^2^)] = 0.081
*wR*(*F* ^2^) = 0.239
*S* = 1.11
3584 reflections
230 parameters
H-atom parameters constrained
Δρ_max_ = 0.44 e Å^−3^
Δρ_min_ = −0.31 e Å^−3^

### 

Data collection: *CAD-4 Software* (Enraf–Nonius, 1989 ▶); cell refinement: *CAD-4 Software*; data reduction: *CORINC* (Dräger & Gattow, 1971 ▶); program(s) used to solve structure: *SIR97* (Altomare *et al.*, 1999 ▶); program(s) used to refine structure: *SHELXL97* (Sheldrick, 2008 ▶); molecular graphics: *PLATON* (Spek, 2009 ▶); software used to prepare material for publication: *PLATON*.

## Supplementary Material

Crystal structure: contains datablocks I, global. DOI: 10.1107/S1600536811019714/bt5556sup1.cif

Structure factors: contains datablocks I. DOI: 10.1107/S1600536811019714/bt5556Isup2.hkl

Supplementary material file. DOI: 10.1107/S1600536811019714/bt5556Isup3.cml

Additional supplementary materials:  crystallographic information; 3D view; checkCIF report

## Figures and Tables

**Fig. 1.**
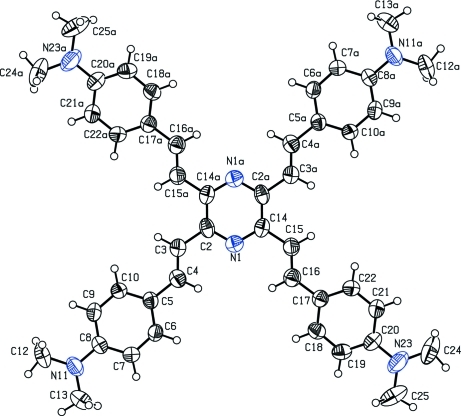
View of compound I. Displacement ellipsoids are drawn at the 50% probability level.

## supplementary crystallographic information

### Comment

The title compound was prepared as a part of a project focusing on star-shaped chromophores  and acidochromic fluorophores, see: Detert & Sugiono (2005), Strehmel *et al.* (2003), and Nemkovich *et al.* (2010). In the crystal, the cruciform adopts a a nearly planar and centrosymmetric conformation. The two linear distyrylpyrazine subunits are not equivalent, one shows small dihedral angles between the aromatic rings and the vinylene groups of -0.3 (5)° (N1—C2—C3—C4) and -177.7 (3)° (C3—C4—C5—C6). The other distyrylpyrazine axis is more distorted. Torsion angles of 8.6°(5) (N1—C14—C15—C16) and -174.4 (3)° (C15—C16—C17—C18) have been measured. Similarly, the torsion angles of 178.2 (3) (C9—C8—N11—C13), -2.2 (5)° (C7—C8—N11—C13) between the the dimethylamino group and the phenylene ring of the more planar arm are significantly smaller than those of the other distyrylpyrazine unit: 156.6 (4)° (C19—C20—N23—C24) and -26.6 (6) (C21—C20—N23—C24). Nevertheless, the strong electronic coupling between all peripheral donors and the central pyrazine acceptor is visible as short C—N bond lengths of the aniline moieties of 1.379 (4)Å for C8—N4 and for C20—N23 and the sums of the bond angles around N11 and N23 of 357 - 358°. These aniline C—N bonds are slightly longer than those found on a linear distyrylpyrazine, (1.368 (2) Å; Fischer *et al.* (2011)), since the acceptor strength of the pyrazine is reduced by two additional donor groups in the title compound. The packing is characterized by sheets of molecules perpendicular to the (101)-direction.

### Experimental

The title compound was prepared by adding potassium *tert*-butylate (9.0 g, 80.4 mmol) in small portions under nitrogen to a cooled (273 K) solution of *p*-*N*,*N*-dimethylaminobenzaldehyde (12.0 g, 80.4 mmol) and tetramethylpyrazine (2.5 g, 18.4 mmol) in DMF (anhyd., 100 ml). Stirring at 273 K was continued for 30 min at 273 K and for 1 week at ambient temperature. Water (200 ml) was added, the precipitate was collected and lower condensation products were extracted in a Soxhlet apparatus with methanol. The residue was recrystallized from methanol/dichloromethane to yield 545 mg (5%) of a dark red solid with m. p. > 670 K.

### Refinement

Hydrogen atoms attached to carbons were placed at calculated positions with C—H = 0.95 Å (aromatic) or 0.98–0.99 Å (*sp*^3^ C-atom). All H atoms were refined in the riding-model approximation with isotropic displacement parameters  set at 1.2–1.5 times of the *U*_eq_ of the parent atom.

### Crystal data

**Table:**

C_44_H_48_N_6_	*F*(000) = 708
*M**_r_* = 660.88	*D*_x_ = 1.159 Mg m^−^^3^
Monoclinic, *P*2_1_/*c*	Cu *K*α radiation, λ = 1.54178 Å
Hall symbol: -P 2ybc	Cell parameters from 3 reflections
*a* = 10.763 (2) Å	θ = 36–47°
*b* = 16.6751 (19) Å	µ = 0.53 mm^−^^1^
*c* = 10.755 (3) Å	*T* = 193 K
β = 101.109 (9)°	Needle, red
*V* = 1894.1 (7) Å^3^	0.30 × 0.20 × 0.10 mm
*Z* = 2	

### Data collection

**Table:**

Enraf–Nonius CAD-4 diffractometer	*R*_int_ = 0.073
Radiation source: rotating anode	θ_max_ = 69.9°, θ_min_ = 4.2°
graphite	*h* = 0→13
ω/2θ scans	*k* = 0→20
3778 measured reflections	*l* = −13→12
3584 independent reflections	3 standard reflections every 60 min
2384 reflections with *I* > 2σ(*I*)	intensity decay: 2%

### Refinement

**Table:**

Refinement on *F*^2^	Primary atom site location: structure-invariant direct methods
Least-squares matrix: full	Secondary atom site location: difference Fourier map
*R*[*F*^2^ > 2σ(*F*^2^)] = 0.081	Hydrogen site location: inferred from neighbouring sites
*wR*(*F*^2^) = 0.239	H-atom parameters constrained
*S* = 1.11	*w* = 1/[σ^2^(*F*_o_^2^) + (0.1104*P*)^2^ + 0.8327*P*] where *P* = (*F*_o_^2^ + 2*F*_c_^2^)/3
3584 reflections	(Δ/σ)_max_ < 0.001
230 parameters	Δρ_max_ = 0.44 e Å^−^^3^
0 restraints	Δρ_min_ = −0.31 e Å^−^^3^

### Special details

**Table:**

Experimental. ^1^H-NMR (CDCl_3_, 400 MHz): δ (ppm) = 7.88 (d, ^3^J = 15.4 Hz, 4H, 2-H ethenyl), 7.58 (d, ^3^J = 8.8 Hz, 8H, 2-H, 6-H phenyl), 7.38 (d, ^3^J = 15.4 Hz, 4H, 1-H ethenyl), 6.75 (d, ^3^J = 8.8 Hz, 8H, 3-H, 6-H phenyl), 3.03 (s, 24H, CH_3_). ^13^C-NMR (CDCl_3_, 75 MHz): δ (ppm) = 150.5 (C-4 phenyl), 144.8 (C-2, C-3, C-4, C-5 pyrazine), 134.8 (C-2 ethenyl), 128.6 (C-3, C-5 phenyl), 125.7 (C-1 phenyl), 118.5 (C-1 ethenyl), 112.2 (C-2, C-6 phenyl), 40.4 (CH_3_). FD-MS: m/z (g/mol) = 220.1 (1.3 %) [M^3+^], 330.2 (10.8 %) [M^2+^], 660.2 (100 %) [M^+^]. IR (ATR): ν = 2852, 1595, 1519, 1430, 1356, 1322, 1153, 965, 946, 799 cm^-1^. UV-Vis (CH_2_Cl_2_): λ_max_ = 448 nm, ε = 41446 cm^2^/mmol. Fluorescence (CH_2_Cl_2_): λ_max_ = 580 nm. HR-ESI-MS (g/mol): Calcd. for C_44_H_48_N_6_: 660.3940, found: 660.3915.
Geometry. All e.s.d.'s (except the e.s.d. in the dihedral angle between two l.s. planes) are estimated using the full covariance matrix. The cell e.s.d.'s are taken into account individually in the estimation of e.s.d.'s in distances, angles and torsion angles; correlations between e.s.d.'s in cell parameters are only used when they are defined by crystal symmetry. An approximate (isotropic) treatment of cell e.s.d.'s is used for estimating e.s.d.'s involving l.s. planes.
Refinement. Refinement of *F*^2^ against ALL reflections. The weighted *R*-factor *wR* and goodness of fit *S* are based on *F*^2^, conventional *R*-factors *R* are based on *F*, with *F* set to zero for negative *F*^2^. The threshold expression of *F*^2^ > σ(*F*^2^) is used only for calculating *R*-factors(gt) *etc*. and is not relevant to the choice of reflections for refinement. *R*-factors based on *F*^2^ are statistically about twice as large as those based on *F*, and *R*- factors based on ALL data will be even larger.

### Fractional atomic coordinates and isotropic or equivalent isotropic displacement parameters (Å^2^)

**Table:**

	*x*	*y*	*z*	*U*_iso_*/*U*_eq_	
N1	0.4285 (2)	0.53184 (16)	0.5798 (2)	0.0526 (7)	
C2	0.4847 (3)	0.57967 (17)	0.5085 (3)	0.0504 (7)	
C3	0.4652 (3)	0.66661 (19)	0.5238 (3)	0.0550 (8)	
H3	0.5045	0.7023	0.4744	0.066*	
C4	0.3983 (3)	0.69750 (19)	0.5996 (3)	0.0546 (8)	
H4	0.3625	0.6602	0.6494	0.066*	
C5	0.3701 (3)	0.78186 (17)	0.6194 (3)	0.0476 (7)	
C6	0.2997 (3)	0.80216 (18)	0.7090 (3)	0.0519 (8)	
H6	0.2713	0.7604	0.7568	0.062*	
C7	0.2687 (3)	0.87950 (19)	0.7326 (3)	0.0524 (8)	
H7	0.2196	0.8901	0.7953	0.063*	
C8	0.3084 (3)	0.94316 (17)	0.6651 (3)	0.0498 (7)	
C9	0.3792 (3)	0.92395 (18)	0.5729 (3)	0.0548 (8)	
H9	0.4075	0.9656	0.5248	0.066*	
C10	0.4085 (3)	0.84511 (18)	0.5509 (3)	0.0504 (7)	
H10	0.4561	0.8337	0.4873	0.060*	
N11	0.2801 (3)	1.02172 (16)	0.6882 (3)	0.0671 (8)	
C12	0.3545 (4)	1.08608 (19)	0.6484 (4)	0.0746 (11)	
H12A	0.4442	1.0776	0.6846	0.112*	
H12B	0.3270	1.1375	0.6779	0.112*	
H12C	0.3424	1.0866	0.5557	0.112*	
C13	0.2042 (3)	1.0400 (2)	0.7805 (4)	0.0731 (11)	
H13A	0.1276	1.0068	0.7649	0.110*	
H13B	0.1804	1.0968	0.7742	0.110*	
H13C	0.2528	1.0289	0.8656	0.110*	
C14	0.4417 (3)	0.45242 (19)	0.5724 (3)	0.0506 (7)	
C15	0.3762 (3)	0.40327 (19)	0.6534 (3)	0.0556 (8)	
H15	0.3938	0.3474	0.6585	0.067*	
C16	0.2959 (3)	0.4313 (2)	0.7183 (3)	0.0564 (8)	
H16	0.2803	0.4874	0.7116	0.068*	
C17	0.2262 (3)	0.38681 (18)	0.8010 (3)	0.0490 (7)	
C18	0.1360 (3)	0.4262 (2)	0.8539 (3)	0.0553 (8)	
H18	0.1227	0.4818	0.8370	0.066*	
C19	0.0648 (3)	0.3888 (2)	0.9297 (3)	0.0595 (8)	
H19	0.0029	0.4184	0.9625	0.071*	
C20	0.0826 (3)	0.3074 (2)	0.9590 (3)	0.0596 (9)	
C21	0.1750 (3)	0.2668 (2)	0.9072 (3)	0.0590 (8)	
H21	0.1897	0.2114	0.9253	0.071*	
C22	0.2444 (3)	0.30536 (19)	0.8310 (3)	0.0546 (8)	
H22	0.3064	0.2761	0.7977	0.065*	
N23	0.0169 (3)	0.2693 (2)	1.0401 (4)	0.0948 (12)	
C24	0.0001 (5)	0.1823 (3)	1.0311 (5)	0.1053 (17)	
H24A	−0.0489	0.1683	0.9474	0.158*	
H24B	−0.0452	0.1640	1.0967	0.158*	
H24C	0.0831	0.1562	1.0433	0.158*	
C25	−0.0672 (4)	0.3125 (3)	1.1036 (4)	0.0988 (16)	
H25A	−0.0253	0.3612	1.1416	0.148*	
H25B	−0.0904	0.2787	1.1702	0.148*	
H25C	−0.1438	0.3271	1.0426	0.148*	

### Atomic displacement parameters (Å^2^)

**Table:**

	*U*^11^	*U*^22^	*U*^33^	*U*^12^	*U*^13^	*U*^23^
N1	0.0507 (14)	0.0585 (16)	0.0480 (14)	−0.0022 (12)	0.0079 (12)	−0.0034 (12)
C2	0.0549 (17)	0.0391 (16)	0.0507 (17)	−0.0038 (13)	−0.0061 (14)	0.0016 (13)
C3	0.0572 (18)	0.0496 (18)	0.0590 (19)	−0.0042 (14)	0.0131 (15)	0.0023 (15)
C4	0.0517 (17)	0.0487 (18)	0.0616 (19)	0.0010 (13)	0.0068 (15)	−0.0002 (15)
C5	0.0466 (15)	0.0420 (15)	0.0532 (17)	−0.0028 (12)	0.0071 (13)	−0.0003 (13)
C6	0.0524 (17)	0.0450 (17)	0.0604 (19)	−0.0010 (13)	0.0163 (15)	0.0080 (14)
C7	0.0548 (17)	0.0517 (17)	0.0539 (18)	0.0016 (14)	0.0189 (14)	−0.0023 (14)
C8	0.0513 (17)	0.0402 (16)	0.0555 (17)	0.0021 (13)	0.0045 (14)	−0.0014 (13)
C9	0.0589 (18)	0.0446 (17)	0.0617 (19)	−0.0071 (14)	0.0134 (15)	0.0078 (14)
C10	0.0514 (17)	0.0476 (17)	0.0551 (18)	−0.0031 (13)	0.0179 (14)	−0.0038 (14)
N11	0.081 (2)	0.0423 (15)	0.078 (2)	0.0051 (13)	0.0160 (17)	−0.0054 (14)
C12	0.097 (3)	0.0389 (18)	0.079 (2)	−0.0035 (17)	−0.006 (2)	0.0002 (16)
C13	0.068 (2)	0.063 (2)	0.083 (3)	0.0145 (17)	0.002 (2)	−0.0182 (19)
C14	0.0511 (16)	0.0507 (18)	0.0476 (16)	−0.0105 (13)	0.0033 (13)	0.0066 (14)
C15	0.0636 (19)	0.0481 (17)	0.0553 (18)	−0.0025 (15)	0.0119 (15)	0.0016 (14)
C16	0.0555 (18)	0.0558 (19)	0.0569 (19)	−0.0053 (15)	0.0086 (15)	−0.0011 (15)
C17	0.0479 (16)	0.0498 (17)	0.0484 (16)	−0.0038 (13)	0.0071 (13)	−0.0003 (13)
C18	0.0544 (18)	0.0520 (18)	0.0596 (19)	0.0074 (14)	0.0112 (15)	0.0000 (15)
C19	0.0494 (17)	0.071 (2)	0.061 (2)	0.0080 (15)	0.0173 (15)	−0.0014 (17)
C20	0.0519 (18)	0.073 (2)	0.0561 (19)	−0.0061 (16)	0.0153 (15)	0.0104 (17)
C21	0.067 (2)	0.0506 (18)	0.061 (2)	−0.0041 (15)	0.0174 (17)	0.0063 (15)
C22	0.0586 (18)	0.0529 (18)	0.0558 (18)	0.0003 (14)	0.0200 (15)	−0.0054 (14)
N23	0.089 (2)	0.107 (3)	0.101 (3)	−0.005 (2)	0.049 (2)	0.031 (2)
C24	0.091 (3)	0.110 (4)	0.114 (4)	−0.020 (3)	0.019 (3)	0.057 (3)
C25	0.062 (2)	0.168 (5)	0.074 (3)	−0.008 (3)	0.031 (2)	0.013 (3)

### Geometric parameters (Å, °)

**Table:**

N1—C2	1.330 (4)	C13—H13C	0.9800
N1—C14	1.336 (4)	C14—C2^i^	1.392 (4)
C2—C14^i^	1.392 (4)	C14—C15	1.471 (4)
C2—C3	1.479 (4)	C15—C16	1.299 (4)
C3—C4	1.294 (4)	C15—H15	0.9500
C3—H3	0.9500	C16—C17	1.470 (4)
C4—C5	1.463 (4)	C16—H16	0.9500
C4—H4	0.9500	C17—C18	1.382 (4)
C5—C6	1.379 (4)	C17—C22	1.401 (4)
C5—C10	1.393 (4)	C18—C19	1.371 (4)
C6—C7	1.368 (4)	C18—H18	0.9500
C6—H6	0.9500	C19—C20	1.398 (5)
C7—C8	1.399 (4)	C19—H19	0.9500
C7—H7	0.9500	C20—N23	1.379 (4)
C8—N11	1.379 (4)	C20—C21	1.404 (5)
C8—C9	1.399 (4)	C21—C22	1.371 (4)
C9—C10	1.383 (4)	C21—H21	0.9500
C9—H9	0.9500	C22—H22	0.9500
C10—H10	0.9500	N23—C25	1.430 (6)
N11—C13	1.434 (5)	N23—C24	1.463 (6)
N11—C12	1.452 (4)	C24—H24A	0.9800
C12—H12A	0.9800	C24—H24B	0.9800
C12—H12B	0.9800	C24—H24C	0.9800
C12—H12C	0.9800	C25—H25A	0.9800
C13—H13A	0.9800	C25—H25B	0.9800
C13—H13B	0.9800	C25—H25C	0.9800
			
C2—N1—C14	119.6 (3)	N1—C14—C2^i^	119.9 (3)
N1—C2—C14^i^	120.5 (3)	N1—C14—C15	116.7 (3)
N1—C2—C3	115.7 (3)	C2^i^—C14—C15	123.5 (3)
C14^i^—C2—C3	123.8 (3)	C16—C15—C14	124.2 (3)
C4—C3—C2	124.6 (3)	C16—C15—H15	117.9
C4—C3—H3	117.7	C14—C15—H15	117.9
C2—C3—H3	117.7	C15—C16—C17	127.9 (3)
C3—C4—C5	129.1 (3)	C15—C16—H16	116.1
C3—C4—H4	115.5	C17—C16—H16	116.1
C5—C4—H4	115.5	C18—C17—C22	116.4 (3)
C6—C5—C10	116.2 (3)	C18—C17—C16	119.3 (3)
C6—C5—C4	119.7 (3)	C22—C17—C16	124.3 (3)
C10—C5—C4	124.1 (3)	C19—C18—C17	123.0 (3)
C7—C6—C5	123.2 (3)	C19—C18—H18	118.5
C7—C6—H6	118.4	C17—C18—H18	118.5
C5—C6—H6	118.4	C18—C19—C20	120.5 (3)
C6—C7—C8	120.6 (3)	C18—C19—H19	119.7
C6—C7—H7	119.7	C20—C19—H19	119.7
C8—C7—H7	119.7	N23—C20—C19	121.7 (3)
N11—C8—C9	121.0 (3)	N23—C20—C21	121.2 (3)
N11—C8—C7	121.8 (3)	C19—C20—C21	117.1 (3)
C9—C8—C7	117.2 (3)	C22—C21—C20	121.3 (3)
C10—C9—C8	120.8 (3)	C22—C21—H21	119.3
C10—C9—H9	119.6	C20—C21—H21	119.3
C8—C9—H9	119.6	C21—C22—C17	121.6 (3)
C9—C10—C5	122.0 (3)	C21—C22—H22	119.2
C9—C10—H10	119.0	C17—C22—H22	119.2
C5—C10—H10	119.0	C20—N23—C25	121.3 (4)
C8—N11—C13	120.2 (3)	C20—N23—C24	119.2 (4)
C8—N11—C12	119.7 (3)	C25—N23—C24	116.7 (4)
C13—N11—C12	117.8 (3)	N23—C24—H24A	109.5
N11—C12—H12A	109.5	N23—C24—H24B	109.5
N11—C12—H12B	109.5	H24A—C24—H24B	109.5
H12A—C12—H12B	109.5	N23—C24—H24C	109.5
N11—C12—H12C	109.5	H24A—C24—H24C	109.5
H12A—C12—H12C	109.5	H24B—C24—H24C	109.5
H12B—C12—H12C	109.5	N23—C25—H25A	109.5
N11—C13—H13A	109.5	N23—C25—H25B	109.5
N11—C13—H13B	109.5	H25A—C25—H25B	109.5
H13A—C13—H13B	109.5	N23—C25—H25C	109.5
N11—C13—H13C	109.5	H25A—C25—H25C	109.5
H13A—C13—H13C	109.5	H25B—C25—H25C	109.5
H13B—C13—H13C	109.5		
			
C14—N1—C2—C14^i^	−0.8 (5)	C2—N1—C14—C2^i^	0.7 (5)
C14—N1—C2—C3	−179.7 (3)	C2—N1—C14—C15	−179.6 (3)
N1—C2—C3—C4	−0.3 (5)	N1—C14—C15—C16	8.6 (5)
C14^i^—C2—C3—C4	−179.2 (3)	C2^i^—C14—C15—C16	−171.8 (3)
C2—C3—C4—C5	−178.2 (3)	C14—C15—C16—C17	179.9 (3)
C3—C4—C5—C6	−177.7 (3)	C15—C16—C17—C18	−174.4 (3)
C3—C4—C5—C10	3.4 (5)	C15—C16—C17—C22	5.5 (5)
C10—C5—C6—C7	−0.6 (5)	C22—C17—C18—C19	−1.4 (5)
C4—C5—C6—C7	−179.5 (3)	C16—C17—C18—C19	178.5 (3)
C5—C6—C7—C8	−0.2 (5)	C17—C18—C19—C20	1.1 (5)
C6—C7—C8—N11	−179.0 (3)	C18—C19—C20—N23	176.6 (3)
C6—C7—C8—C9	0.6 (4)	C18—C19—C20—C21	−0.3 (5)
N11—C8—C9—C10	179.3 (3)	N23—C20—C21—C22	−177.1 (3)
C7—C8—C9—C10	−0.3 (5)	C19—C20—C21—C22	−0.2 (5)
C8—C9—C10—C5	−0.5 (5)	C20—C21—C22—C17	−0.2 (5)
C6—C5—C10—C9	0.9 (4)	C18—C17—C22—C21	0.9 (5)
C4—C5—C10—C9	179.8 (3)	C16—C17—C22—C21	−178.9 (3)
C9—C8—N11—C13	178.2 (3)	C19—C20—N23—C25	−3.6 (6)
C7—C8—N11—C13	−2.2 (5)	C21—C20—N23—C25	173.2 (4)
C9—C8—N11—C12	−19.6 (5)	C19—C20—N23—C24	156.6 (4)
C7—C8—N11—C12	160.1 (3)	C21—C20—N23—C24	−26.6 (6)

Symmetry codes: (i) −*x*+1, −*y*+1, −*z*+1.
